# The role of peripheral β-amyloid in insulin resistance, insulin secretion, and prediabetes: *in vitro* and population-based studies

**DOI:** 10.3389/fendo.2023.1195658

**Published:** 2023-07-19

**Authors:** Zihui Xu, Juan Chen, Pei Wang, Linyan Li, Shan Hu, Hongjie Liu, Yue Huang, Xiaoxing Mo, Hong Yan, Zhilei Shan, Di Wang, Jian Xu, Liegang Liu, Xiaobo Peng

**Affiliations:** ^1^Department of Nutrition and Food Hygiene, Hubei Key Laboratory of Food Nutrition and Safety, School of Public Health, Tongji Medical College, Huazhong University of Science & Technology, Wuhan, China; ^2^Ministry of Education Key Lab of Environment and Health, School of Public Health, Tongji Medical College, Huazhong University of Science & Technology, Wuhan, China; ^3^Xiangyang Key Laboratory of Public Health and Epidemic Prevention Materials Research, Xiangyang Public Inspection and Testing Center, Xiangyang, China; ^4^Department of Elderly Health Management, Shenzhen Center for Chronic Disease Control, Shenzhen, China

**Keywords:** peripheral β-amyloid, *in vitro* study, insulin resistance, insulin secretion, prediabetes, population-based study

## Abstract

**Background:**

Previous experimental studies have shown that mice overexpressing amyloid precursor protein, in which β-amyloid (Aβ) is overproduced, exhibit peripheral insulin resistance, pancreatic impairment, and hyperglycemia. We aimed to explore the effects of Aβ on insulin action and insulin secretion *in vitro* and the association of plasma Aβ with prediabetes in human.

**Methods:**

We examined the effects of Aβ40 and Aβ42 on insulin-inhibited glucose production in HepG2 cells, insulin-promoted glucose uptake in C2C12 myotubes, and insulin secretion in INS-1 cells. Furthermore, we conducted a case-control study (N = 1142) and a nested case-control study (N = 300) within the prospective Tongji-Ezhou cohort. Odds ratios (ORs) and 95% confidence intervals (CIs) for prediabetes were estimated by using conditional logistic regression analyses.

**Results:**

In the *in vitro* studies, Aβ40 and Aβ42 dose-dependently attenuated insulin-inhibited glucose production in HepG2 cells, insulin-promoted glucose uptake in C2C12 myotubes, and basal and glucose-stimulated insulin secretion in INS-1 cells. In the case-control study, plasma Aβ40 (adjusted OR: 2.00; 95% CI: 1.34, 3.01) and Aβ42 (adjusted OR: 1.94; 95% CI: 1.33, 2.83) were positively associated with prediabetes risk when comparing the extreme quartiles. In the nested case-control study, compared to the lowest quartile, the highest quartile of plasma Aβ40 and Aβ42 were associated with 3.51-fold (95% CI: 1.61, 7.62) and 2.75-fold (95% CI: 1.21, 6.22) greater odds of prediabetes, respectively.

**Conclusion:**

Elevated plasma Aβ40 and Aβ42 levels were associated with increased risk of prediabetes in human subjects, which may be through impairing insulin sensitivity in hepatocytes and myotubes and insulin secretion in pancreatic β-cells.

## 1 Introduction

β-amyloid (Aβ), mainly including Aβ40 and Aβ42, is a natural product from enzymatic proteolysis of amyloid precursor protein (APP) and has been found to be deposited in the brains of Alzheimer’s disease patients (1). Notably, Aβ is widely generated in the brain as well as various peripheral tissues (2–5). Accumulating evidence indicates that peripheral Aβ involves in regulating metabolism, especially glucose and insulin metabolism. Previous animal studies have demonstrated that mice overexpressing APP, in which Aβ is overproduced, exhibit peripheral insulin resistance and pancreatic impairment (6, 7). In addition, Aβ deposition has been found in human peripheral tissues taking part in glucose regulation, including skeletal muscle, liver, and pancreas (8–11). However, the effects of Aβ40 and Aβ42 on these peripheral tissues remain unclear.

Prediabetes, defined as an intermediate condition between normoglycemia and diabetes, is mainly manifested as peripheral insulin resistance and β-cell dysfunction (12). There is increasing number of individuals with prediabetes worldwide, with approximated 470 million people suffering from prediabetes by 2030 (13). Prediabetes individuals have been reported to exert greater risk of type 2 diabetes (T2D), cardiovascular disease (CVD), and mortality later in life (14, 15). Previous animal studies found that APP transgenic mice with high plasma Aβ concentrations displayed a prediabetes phenotype (16, 17). Yet, few epidemiological studies have explored the relationship between plasm Aβ and prediabetes.

Therefore, we examined the effects of Aβ40 and Aβ42 on insulin sensitivity in hepatocytes and myotubes as well as insulin secretion in pancreatic β-cells via *in vitro* studies. Furthermore, we evaluated the associations between plasma Aβ40 and Aβ42 and prediabetes in two independent populations, including a case-control study and a nested case-control study within the prospective Tongji-Ezhou cohort.

## 2 Materials and methods

### 2.1 Cell culture and Aβ treatment

HepG2 (human hepatoma) cell line was purchased from ATCC (Rockville, MD, USA); C2C12 (murine myoblast) and INS-1 (rat insulinoma) cell lines were purchased from the Type Culture Collection of Chinese Academy of Sciences (Shanghai, China). All reagents were purchased from Gibco (Grand Island, NY, USA) unless otherwise mentioned. We cultured HepG2 cells in minimal essential medium supplemented with 10% (vol./vol.) fetal bovine serum (FBS), 1% (vol./vol.) non-essential amino acids, 1 mmol/l sodium pyruvate, and 1% (vol./vol.) penicillin-streptomycin. We maintained C2C12 cells in Dulbecco’s modified Eagle medium (DMEM) supplemented with 10% (vol./vol.) FBS and 1% (vol./vol.) penicillin-streptomycin, and differentiated the cells into myotubes in the medium supplemented with 2% (vol./vol.) horse serum for 4 days when they reached confluence. We cultured INS-1 cells in RPMI 1640 medium supplemented with 10% (vol./vol.) FBS, 10 mmol/l HEPES, 1 mmol/l sodium pyruvate, 50 μmol/l 2-mercaptoethanol (Genom, Hangzhou, China), and 1% (vol./vol.) penicillin-streptomycin. All cells were cultivated in a humidified condition (37 °C, 5% CO2).

Synthetic Aβ40, Aβ42, Aβ40-1, and Aβ42-1 (Chinapeptides, Shanghai, China) were dissolved in dimethyl sulfoxide at 5 mmol/l and then diluted with phosphate buffered saline at 400 μmol/l. They were further incubated at 37 °C, 220 rev/min for 48 h before use. Considering the deposition of Aβ in human peripheral tissues (8–11), Aβ concentrations in peripheral tissues may greatly exceed that in plasma. We treated cells with different doses (0, 2, 10, and 20 μmol/l) of Aβ40 or Aβ42 according to previous studies (17–20). In addition, Aβ40-1 and Aβ42-1 were used as control peptides with reverse sequence, and cells were treated with 20 μmol/l Aβ40-1 or Aβ42-1.

### 2.2 Cell viability assay

Cell viability of various cell lines was detected by CCK-8 (Dojindo, Kyushu Island, Japan). HepG2 cells, C2C12 myotubes, and INS-1 cells in 96-well plates were treated with different doses (0, 2, 10, and 20 μmol/l) of Aβ40 or Aβ42 for different periods of time (12, 24, and 48 h). Next, we added 10 μl CCK-8 reagent per well and measured optical density values by a microplate reader (Tecan, Männedorf, Switzerland) at 450 nm.

### 2.3 Glucose production assay

We performed glucose production assay according to the method described previously (21). We seeded HepG2 cells in 12-well plate and treated the cells with different doses of Aβ for 48 h. After treatment, we further incubated the cells with the medium for glucose production (glucose-free DMEM containing 2 mmol/l sodium pyruvate and 20 mmol/l sodium lactate) without or with 100 nmol/l insulin. After 3 h, the glucose level of supernatant was measure by a fluorescent glucose assay kit (Invitrogen, Carlsbad, CA, USA) and normalized to total protein amount.

### 2.4 Glucose uptake assay

Glucose uptake in C2C12 myotubes was determined as previously described (22). C2C12 myotubes in 12-well plate were treated with different doses of Aβ for 48 h. Next, C2C12 myotubes were glucose starved in glucose-free DMEM for 1 h and further incubated in phenol red-free DMEM supplemented without or with 100 nmol/l insulin for 2 h. Subsequently, we detected the glucose level of supernatant with the use of glucose assay kit (Invitrogen). Glucose intake was calculated by subtracting the remaining medium glucose from the fresh medium glucose and further normalized to the total protein amount.

### 2.5 Insulin secretion assay

We conducted insulin secretion assay according to the method described previously (23). We seeded INS-1 cells in 12-well plate and treated the cells with different doses of Aβ for 24 h. After treatment, the cells were glucose starved in the glucose-free RPMI 1640 media for 2 h and then incubated in the glucose-free RPMI 1640 media supplemented with 3 mmol/l or 15 mmol/l glucose for 2 h. After 2 h, the insulin level of supernatant was determined with a rat insulin assay kit (Mercodia, Uppsala, Sweden), and total protein amount was quantified to normalize insulin values.

### 2.6 Study design and population

The initial case-control study included 1142 participants (571 newly diagnosed prediabetes cases and 571 healthy controls) in Wuhan, China. We recruited cases from patients for the first time diagnosed as prediabetes in Tongji Hospital from 2012 to 2015. Concurrently, healthy controls were recruited from a general population receiving a regular medical examination in Tongji Hospital and matched 1:1 to cases on basis of age ( ± 3 years) and gender. In this study, we included participants who were aged ≥30 and ≤80 years, body mass index (BMI) <40 kg/m2, no previous diagnosis of prediabetes or diabetes, and no use of antihyperlipidemic medication. Additionally, patients diagnosed as any other clinically systemic illness, infectious disease, chronic inflammatory illness, or acute disease were excluded.

To clarify the prospective association of plasma Aβ concentration with risk of prediabetes, we further conducted a nested case-control study within the Tongji-Ezhou cohort, a prospective cohort of 5533 participants from Echeng Stell, including 3101 retired employees and 2432 in-service employees. The cohort enrollment period was 2013–2015, and the first follow-up survey was completed by mid-2020. We conducted this study within the sub cohort of retired employees because the incidence rate of prediabetes was low in young in-service employees. At the first follow-up, 119 retired employees were diagnosed as new-onset prediabetes cases by detecting fasting plasma glucose (FPG). According to the same inclusion criteria as the case-control study, we excluded 2 new-onset prediabetes cases with age >80 years. We also excluded 17 new-onset prediabetes cases without adequate blood samples. Two control participants were matched to each case on basis of age ( ± 3 years) and gender from retired employees without prediabetes or diabetes. Eventually, 100 cases and 200 age- and gender-matched controls were included for analysis.

These two population-based studies were approved by the Ethics Committee of Tongji Medical College and conducted by complying with the Declaration of Helsinki. Written informed consent was provided by the participants included in the study.

### 2.7 Assessment of prediabetes

In the case-control study, the definition of prediabetes was in accordance with the 1999 WHO criteria (24): FPG ≥6.1 and <7.0 mmol/l or 2-h oral glucose tolerance test (OGTT) ≥7.8 and <11.1 mmol/l. In the Tongji cohort, new-onset prediabetes was diagnosed when FPG was ≥6.1 and <7.0 mmol/l.

### 2.8 Assessment of covariates

Sociodemographic characteristics of all participants, including age, gender, family history of diabetes, lifestyle habits (alcohol drinking, smoking, and physical activity), and history of diseases (CVD and hypertension) were obtained from a standardized questionnaire. The definition of physical activity was regular exercise for more than 60min/week in the past 6 months. The trained staff measured weight (kg) and height (m) and calculated BMI by dividing weight by the square of height. We determined fasting plasma insulin (FPI), FPG, total cholesterol, triacylglycerols, HDL-cholesterol (HDL-C), and LDL-cholesterol (LDL-C) using fasting plasma samples as previously described (25). 2-h OGTT plasma samples were collected from subjects who were enrolled from Tongji Hospital and used to determine 2-h post-glucose load values. We calculated the index of homeostasis model assessment of insulin resistance (HOMA-IR) and β-cell function (HOMA-β) according to the following equations: HOMA-IR = FPI (pmol/l) × FPG (mmol/l) ÷ 156.3; HOMA-β = 2.88 × FPI (pmol/l) ÷ [FPG (mmol/l) - 3.5]. The triglyceride-glucose (TyG) index was calculated as: ln [FPG (mg/dl) × fasting TG (mg/dl) ÷ 2].

### 2.9 Measurement of plasma Aβ levels

We simultaneously determined Aβ40 and Aβ42 levels in fasting plasma with the use of validated assay kits (Meso Scale Discovery, Rockville, USA). Both the within- and between-assay coefficients of variation of plasma Aβ40 and Aβ42 were <10%. Plasma Aβ levels of the included participants were above the limit of detection (Aβ40: 20.0 ng/l; Aβ42: 2.5 ng/l). The assays were performed by investigators who were blind to prediabetes status.

### 2.10 Statistical analysis

For the *in vitro* studies, all data are expressed as mean ± standard error of mean (SEM) of three independent experiments. We assessed the differences among groups using one-way analysis of variance and further conducted multiple comparisons by least significant difference test.

For the population-based studies, characteristics of prediabetes cases and control subjects were compared using *t*-test, Mann–Whitney *U* test, or *χ*^2^ test. We further evaluated the correlation between plasma Aβ and the variables of interest with Spearman correlation test. ORs and 95% CIs for prediabetes were estimated by conditional logistic regression analyses, in which plasma Aβ40 and Aβ42 levels were classified into quartiles according the distribution in control group or as continuous variables presented as an increment by 30 ng/l (Aβ40) or 5 ng/l (Aβ42). Adjustments were made for several potential confounders, including age (years), gender (female or male), BMI (<18.5, 18.5–23.9, 24–27.9, or ≥28 kg/m2), family history of diabetes (yes or no), alcohol drinking habit (current, former, or never), smoking habit (current, former, or never), physical activity (yes or no), CVD (yes or no), and hypertension (yes or no). We conducted the linear trend test by assigning each quartile with the median of plasma Aβ40 or Aβ42 and using it as a continuous variable. Receiver-operating characteristic (ROC) curves were plotted to estimate the prediction of plasma Aβ levels on prediabetes. We compared area under the curves (AUCs) of model 1 including traditional risk factors (age, gender, BMI, family history of diabetes, alcohol drinking habit, smoking habit, physical activity, CVD, hypertension, FPG, and FPI) and model 2 with plasma Aβ40 and Aβ42 levels further added in.

All data analyses were conducted with the use of SPSS 20.0 (SPSS Inc., Chicago, IL) and R 4.2.2 (The R Foundation, http://www.r-project.org). All presented *p* values are 2-tailed and considered as significance at level of 0.05.

## 3 Results

### 3.1 *In vitro* studies

Cells were treated with different doses of Aβ40 and Aβ42, and viability was detected at 12, 24, and 48 h. Exposure to 20 μmol/l Aβ40 and Aβ42 induced a significant decrease in cell viability for all cell lines (**Supplemental Figures 1–3**). The viability of HepG2 cells and C2C12 myotubes were still above 80% after exposure to 20 μmol/l Aβ40 and Aβ42 for 48 h. In INS-1 cells treated with 20 μmol/l Aβ40 and Aβ42 for 48 h, cell viability decreased to 67% and 77%, respectively. To maintain high cell viability (>80%), INS-1 cells were treated with various doses of Aβ40 and Aβ42 for 24 h in insulin secretion assay. To rule out the effects of cytotoxicity of Aβ on subsequent experiments, the amounts of glucose production, glucose uptake, and insulin secretion were normalized to total protein amount of the remaining cells in the culture plate.

**Figure 1:**
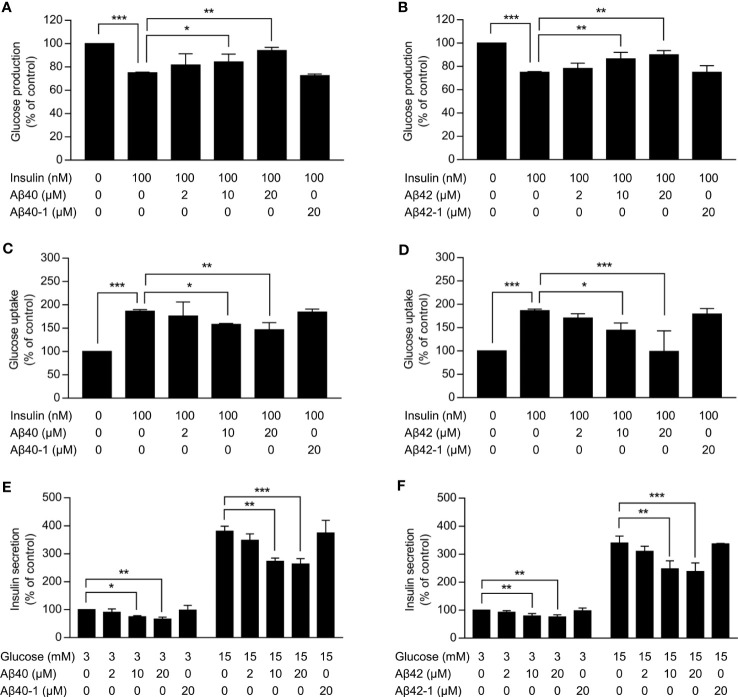
Effects of Aβ on insulin sensitivity in hepatocytes and myotubes and insulin secretion in pancreatic β-cells. **(A,B)** Glucose production in HepG2 cells treated with the indicated concentrations of Aβ40 or Aβ40-1 **(A)** or Aβ42 or Aβ42-1(b) for 48 h and then incubated without or with 100 nmol/l insulin for 3 h (n = 3). **(C, D)** Glucose uptake in C2C12 myotubes treated with the indicated concentrations of Aβ40 or Aβ40-1 **(C)** or Aβ42 or Aβ42-1(D) for 48 h and glucose starved for 1 h, and then incubated without or with 100 nmol/l insulin for 2 h (n = 3). **(E, F)** Insulin secretion in INS-1 cells treated with the indicated concentrations of Aβ40 or Aβ40-1 **(E)** or Aβ42 or Aβ42-1(F) for 24 h and glucose starved for 2 h, then incubated with 3 mmol/l or 15 mmol/l glucose for 2 h (n = 3). Data are presented as mean ± SEM. ^*^p <0.05, ^**^p <0.01, and ^***^p <0.001 for indicated comparisons.

As expected, insulin significantly inhibited the glucose production in HepG2 cells and promoted the glucose uptake in C2C12 myotubes (*p <*0.001) (**Figures 1A-D**). Aβ40 and Aβ42 dose-dependently attenuated the inhibitory effect of insulin on the glucose production in HepG2 cells (**Figures 1A, B**). The promotional effect of insulin on the glucose uptake in C2C12 myotubes gradually decline with increasing dose of Aβ40 and Aβ42 (**Figures 1C, D**). In addition, Aβ40 and Aβ42 dose-dependently suppressed the basal and glucose-stimulated insulin secretion in INS-1 cells (**Figures 1E, F**). Conversely, neither Aβ40-1 nor Aβ42-1 affected the insulin sensitivity in HepG2 cells and C2C12 myotubes and insulin secretion in INS-1 cells (**Figures 1A-F**).

### 3.2 Case-control study

General characteristics of the 1142 participants (571 newly-diagnosed prediabetes cases and 571 matched controls) are described in **Table 1**. Prediabetes cases showed higher BMI, higher levels of FPI, FPG, triglyceride, LDL-C, HOMA-IR and TyG index as well as lower levels of HDL-C and HOMA-β compared with control subjects. A higher proportion of prediabetes cases had a family history of diabetes, CVD, and hypertension. Additionally, prediabetes cases exhibited higher plasma Aβ40 and Aβ42 levels compared to control subjects.

**Table 1:** Characteristics of the newly diagnosed prediabetes cases and matched controls in the case-control study.

Characteristics	Prediabetes (*n* = 571)	Control (*n* = 571)	*p*
Age (years)	51.46 (11.18)	51.87 (11.08)	0.525
Gender (male), *n* (%)	376 (65.85)	376 (65.85)	1.000
BMI (kg/m^2^)	24.85 (3.30)	23.63 (3.10)	<0.001
Family history of diabetes, *n* (%)	78 (13.66)	42 (7.36)	<0.001
Current drinker, n (%)	172 (30.12)	177 (30.40)	0.748
Current smoker, n (%)	176 (30.82)	206 (36.08)	0.060
Regular physical activity, n (%)	214 (37.48)	233 (40.81)	0.249
CVD, *n* (%)	55 (9.63)	32 (5.60)	0.010
Hypertension, *n* (%)	178 (31.17)	115 (20.14)	<0.001
FPG (mmol/l)	6.33 (6.14–6.61)	5.49 (5.18–5.78)	<0.001
FPI (pmol/l)	64.91 (46.18–92.33)	50.57 (34.76–72.99)	<0.001
HOMA-IR	2.63 (1.86–3.67)	1.75 (1.21–2.58)	<0.001
HOMA-β	67.27 (46.97–101.05)	77.36 (52.12–110.44)	0.011
Triglyceride (mmol/l)	1.49 (1.00–2.39)	1.29 (0.91–1.70)	<0.001
Total cholesterol (mmol/l)	4.76 (4.12–5.46)	4.62 (4.10–5.19)	0.054
LDL-C (mmol/l)	2.57 (1.85–3.25)	2.44 (1.78–3.03)	0.014
HDL-C (mmol/l)	1.29 (1.03–1.51)	1.34 (1.19–1.51)	<0.001
TyG index	8.93 (8.53–9.37)	8.63 (8.26–8.91)	<0.001
Aβ40 (ng/l)	134.09 (118.72–153.59)	127.68 (114.58–145.10)	<0.001
Aβ42 (ng/l)	13.25 (10.78–16.58)	12.27 (10.19–14.92)	<0.001

Values are means (standard deviations) for parametrically distributed data, medians (interquartile ranges) for nonparametrically distributed data, or n (%) for categorical data.

Plasma Aβ40 and Aβ42 were significantly relevant to several parameters of glucose metabolism among healthy participants (**Supplemental Table 1**). After adjustment for age, gender, BMI, family history of diabetes, alcohol drinking habit, smoking habit, physical activity, CVD, and hypertension, plasma Aβ40 was significantly related to FPG (*r* = 0.220), FPI (*r* = 0.128), HOMA-IR (*r* = 0.175), and TyG index (*r* = 0.204). Plasma Aβ42 was also significantly correlated with FPG (*r* = 0.145), HOMA-IR (*r* = 0.114), and TyG index (*r* = 0.106) after these adjustments.

**Table 2** presents odds of newly-diagnosed prediabetes associated with plasma Aβ40 and Aβ42 levels. In multivariate-adjusted analyses, the ORs of prediabetes were 2.00 (95% CI: 1.34, 3.01) for plasma Aβ40 and 1.94 (95% CI: 1.33, 2.83) for plasma Aβ42 when comparing the extreme quartiles. The adjusted ORs for prediabetes were 1.19 (95% CI: 1.03, 1.38) for every 30 ng/l higher plasma Aβ40 and 1.41 (95% CI: 1.20, 1.65) for every 5 ng/l higher plasma Aβ42.

**Table 2:** ORs (95% CIs) for newly diagnosed prediabetes associated with plasma Aβ40 and Aβ42 concentrations in the case-control study.

Variable	Quartiles of plasma Aβ concentrations				*p* for trend	Continuous^b^
	Q1 (lowest)	Q2	Q3	Q4 (highest)		
Plasma Aβ40						
Range (ng/l)	<114.52	114.52–<127.69	127.69–<145.13	≥145.13		
Median (ng/l)	106.30	121.14	134.54	164.86		
Case/control, *n*	105/143	115/143	159/143	192/142		
Model^a^						
Crude	1.00 (ref.)	1.13 (0.78, 1.62)	1.55 (1.10, 2.19)	2.07 (1.43, 2.99)	<0.001	1.21 (1.06, 1.37)
Model 1	1.00 (ref.)	1.13 (0.77, 1.66)	1.57 (1.09, 2.24)	2.10 (1.43, 3.09)	<0.001	1.22 (1.06, 1.40)
Model 2	1.00 (ref.)	1.10 (0.74, 1.63)	1.53 (1.05, 2.24)	2.00 (1.34, 3.01)	<0.001	1.19 (1.03, 1.38)
Plasma Aβ42						
Range (ng/l)	<10.19	10.19–<12.28	12.28–<14.95	≥14.95		
Median (ng/l)	8.70	11.28	13.44	17.69		
Case/control, *n*	116/143	109/142	140/144	206/142		
Model^a^						
Crude	1.00 (ref.)	0.96 (0.68, 1.36)	1.25 (0.89, 1.76)	1.91 (1.35, 2.69)	<0.001	1.41 (1.22, 1.63)
Model 1	1.00 (ref.)	1.01 (0.70, 1.46)	1.37 (0.96, 1.97)	2.00 (1.40, 2.87)	<0.001	1.42 (1.22, 1.65)
Model 2	1.00 (ref.)	0.93 (0.64, 1.37)	1.26 (0.86, 1.85)	1.94 (1.33, 2.83)	<0.001	1.41 (1.20, 1.65)

a Model 1 was adjusted for age, gender, and BMI. Model 2 was additionally adjusted for family history of diabetes, alcohol drinking habit, smoking habit, physical activity, CVD, and hypertension.

b ORs (95% CIs) of newly diagnosed prediabetes associated with each 30 ng/l increase in plasma Aβ40 and each 5 ng/l increase in plasma Aβ42.

Q, quartile; ref., reference.

### 3.3 Nested case-control study

The baseline characteristics of all subjects, including 100 incident prediabetes cases and 200 matched controls, are described in **Table 3**. Individuals who subsequently developed prediabetes had higher FPG, FPI, triglyceride, HOMA-IR, TyG index, and plasma Aβ40 and Aβ42 levels compared to control subjects.

**Table 3:** Baseline characteristic of the incident prediabetes cases and matched controls in the nested case-control study.

Characteristics	Prediabetes (*n* = 100)	Control (*n* = 200)	*p*
Age (years)	62.40 (5.94)	62.39 (5.80)	0.983
Gender (male), *n* (%)	63 (63.00)	126 (63.00)	1.000
BMI (kg/m^2^)	24.11 (2.93)	23.59 (2.76)	0.128
Family history of diabetes, *n* (%)	7 (7.00)	8 (4.00)	0.261
Current drinker, n (%)	31 (31.00)	59 (29.50)	0.789
Current smoker, n (%)	25 (25.00)	66 (33.00)	0.155
Regular physical activity, n (%)	57 (57.00)	106 (53.00)	0.512
Hypertension, *n* (%)	31 (31.00)	54 (27.00)	0.469
CVD, *n* (%)	10 (10.00)	20 (10.00)	1.000
FPG (mmol/l)	5.54 (5.15–5.78)	5.31 (4.99–5.62)	0.003
FPI (pmol/l)	56.95 (40.42–80.16)	48.75 (33.70–67.64)	0.022
HOMA-IR	2.05 (1.37–2.78)	1.69 (1.09–2.37)	0.008
HOMA-β	87.17 (62.23–121.00)	81.33 (56.84–115.78)	0.416
Triglyceride (mmol/l)	1.40 (0.98–1.84)	1.15 (0.82–1.58)	0.004
Total cholesterol (mmol/l)	4.72 (4.30–5.23)	4.85 (4.10–5.40)	0.912
LDL-C (mmol/l)	2.79 (2.38–3.44)	2.86 (2.33–3.37)	0.849
HDL-C (mmol/l)	1.37 (1.16–1.61)	1.38 (1.19–1.60)	0.611
TyG index	8.70 (8.35–8.96)	8.46 (8.13–8.85)	0.001
Aβ40 (ng/l)	143.98 (124.22–174.89)	130.36 (113.03–150.65)	<0.001
Aβ42 (ng/l)	13.96 (12.12–17.31)	12.42 (10.81–14.88)	<0.001

Values are means (standard deviations) for parametrically distributed data, medians (interquartile ranges) for nonparametrically distributed data, or n (%) for categorical data.

**Table 4** presents odds of incident prediabetes associated with plasma Aβ40 and Aβ42 levels. In multivariable analyses, compared to the lowest quartile, the highest quartile of plasma Aβ40 and Aβ42 were associated with 3.51-fold (95% CI: 1.61, 7.62) and 2.75-fold (95% CI: 1.21, 6.22) greater odds of prediabetes, respectively. Every 30 ng/l of plasma Aβ40 and every 5 ng/l of plasma Aβ42 were associated with 56% (95% CI: 24%, 96%) and 60% (95% CI: 17%, 119%) increased odds of prediabetes, respectively.

**Table 4:** ORs (95% CIs) for incident prediabetes associated with plasma Aβ40 and Aβ42 concentrations in the nested case-control study.

Variable	Quartiles of plasma Aβ concentrations				*p* for trend	Continuous^b^
	Q1 (lowest)	Q2	Q3	Q4 (highest)		
Plasma Aβ40						
Range (ng/l)	<113.02	113.02–<130.37	130.37–<150.68	≥150.68		
Median (ng/l)	101.06	121.63	140.96	175.97		
Case/control, n	13/50	20/50	23/50	44/50		
Modela						
Crude	1.00 (ref.)	1.51 (0.67, 3.38)	1.78 (0.78, 4.03)	3.25 (1.56, 6.79)	0.001	1.50 (1.21, 1.87)
Model 1	1.00 (ref.)	1.47 (0.65, 3.33)	1.71 (0.75, 3.94)	3.37 (1.60, 7.08)	<0.001	1.53 (1.23, 1.92)
Model 2	1.00 (ref.)	1.49 (0.64, 3.49)	1.92 (0.81, 4.54)	3.51 (1.61, 7.62)	0.001	1.56 (1.24, 1.96)
Plasma Aβ42						
Range (ng/l)	<10.82	10.82–<12.42	12.42–<14.91	≥14.91		
Median (ng/l)	9.24	11.71	13.49	17.77		
Case/control, n	14/50	16/50	34/50	36/50		
Modela						
Crude	1.00 (ref.)	1.24 (0.53, 2.93)	2.74 (1.25, 6.01)	2.85 (1.32, 6.15)	0.003	1.58 (1.17, 2.12)
Model 1	1.00 (ref.)	1.19 (0.49, 2.86)	2.73 (1.24, 6.00)	2.84 (1.30, 6.21)	0.003	1.57 (1.16, 2.13)
Model 2	1.00 (ref.)	1.17 (0.47, 2.90)	2.61 (1.16, 5.90)	2.75 (1.21, 6.22)	0.006	1.60 (1.17, 2.19)

a Model 1 was adjusted for age, gender, and BMI. Model 2 was additionally adjusted for family history of diabetes, alcohol drinking habit, smoking habit, physical activity, CVD, and hypertension.

b ORs (95% CIs) of incident prediabetes associated with each 30 ng/l increase in plasma Aβ40 and each 5 ng/l increase in plasma Aβ42.

Q, quartile; ref., reference.

**Figure 2:**
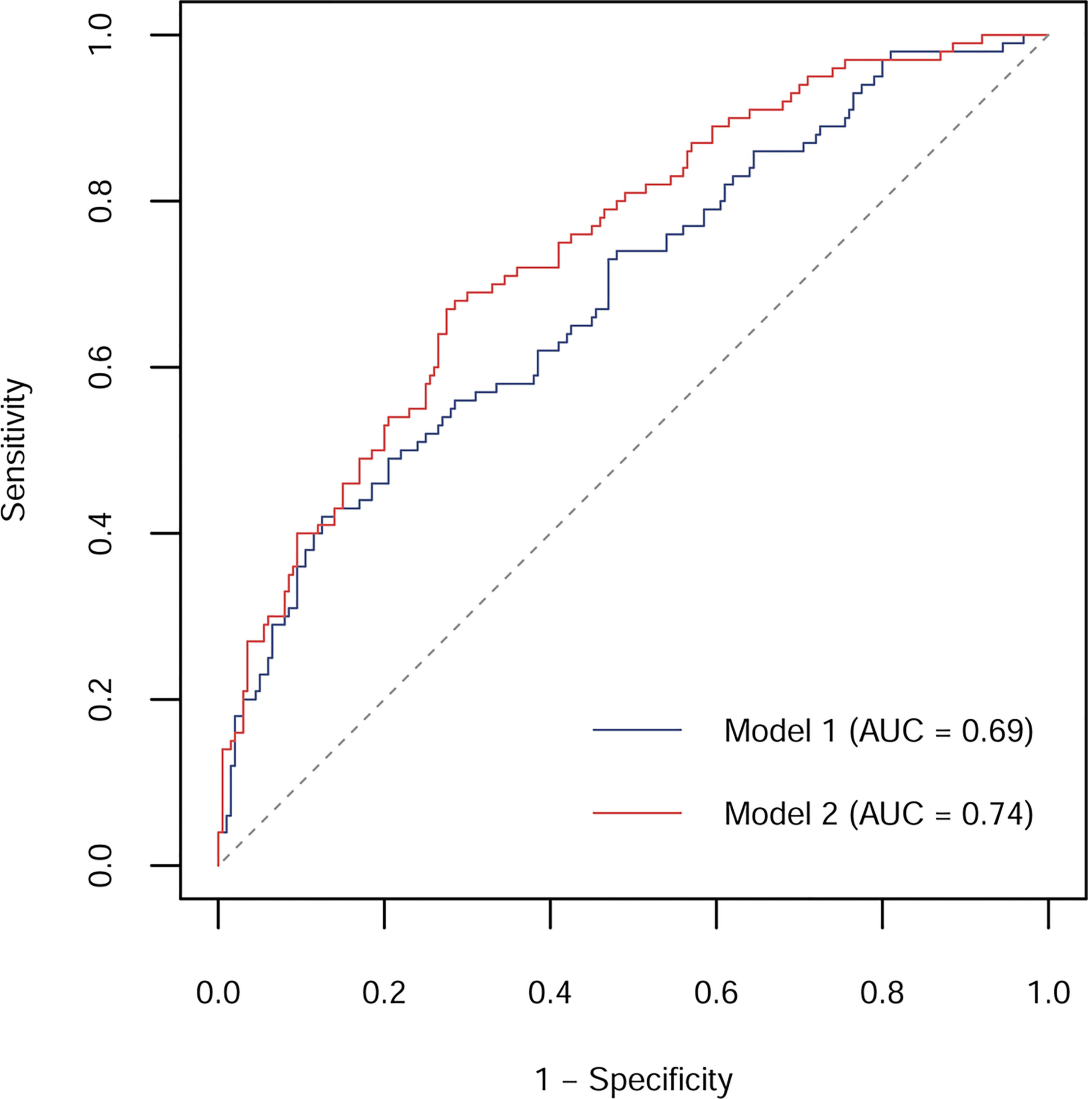
ROC curves and corresponding AUCs for prediction of incident prediabetes by models without or with plasma Aβ40 and Aβ42 in the nested case-control study. Model 1 included age, gender, BMI, family history of diabetes, alcohol drinking habit, smoking habit, physical activity, CVD, hypertension, FPG, and FPI; Model 2 included Model 1 plus plasma Aβ40 and Aβ42.

The AUC for prediction of incident prediabetes was 0.69 (95% CI: 0.62, 0.75) by Model 1, including age, gender, BMI, family history of diabetes, alcohol drinking habit, smoking habit, physical activity, CVD, hypertension, FPG, and FPI (**Figure 2**). The AUC was markedly improved to 0.74 (95% CI: 0.69, 0.80; p = 0.018 for the comparison) when plasma Aβ40 and Aβ42 were added to the model (Model 2).

## 4 Discussion

In the *in vitro* studies, Aβ40 and Aβ42 dose-dependently attenuated insulin-inhibited glucose production in HepG2 cells and insulin-promoted glucose uptake in C2C12 myotubes. Meanwhile, Aβ40 and Aβ42 dose-dependently inhibited the basal and glucose-stimulated insulin secretion in INS-1 cells. In the cross-sectional and prospective population-based studies, we consistently found that elevated plasma Aβ40 and Aβ42 levels were associated with higher odds of prediabetes. Additionally, plasma Aβ40 and Aβ42 significantly improved the predictive value for prediabetes.

It has been reported that *APP* transgenic mice with high plasma Aβ40 and Aβ42 concentrations exhibited peripheral insulin resistance (17, 26). Peripheral insulin resistance in the mice was ameliorated by the active immunity and passive immunity for Aβ (26, 27). Similarly, mice deficient in BACE1, a hydrolase for APP, showed decreased Aβ production and improved insulin resistance when fed a high-fat diet (28). These experimental studies suggest Aβ as a negative regulator of peripheral insulin sensitivity. The current study found positive relevance of plasma Aβ40 and Aβ42 to HOMA-IR and TyG index in healthy subjects. Meanwhile, we observed that both Aβ40 and Aβ42 attenuated insulin action on hepatocytes and myotubes. Our findings indicate that Aβ may promote peripheral insulin resistance in human by directly impairing insulin signaling in liver and skeletal muscle. Supporting our findings, Zhang and colleagues discovered that Aβ could impair hepatic insulin signaling via activating Janus kinase 2 (17, 27). In addition, Aβ has also been reported to impair neuronal insulin signaling through competing for insulin binding to the insulin receptor (29), removing insulin receptors from neuronal surface (30, 31) and activating c-Jun N-terminal kinase to trigger insulin receptor substrate-1 inhibition (32, 33). But whether these mechanisms are involved in Aβ-induced insulin resistance in peripheral tissues needs to be further explored.

Previous study, using gene regulation network analyses, identified *APP* as a top candidate gene for the regulation of insulin secretion from pancreatic islets (34). Meanwhile, the study found that knockout of *APP* in mice enhanced insulin secretion from pancreatic islets through an unclear mechanism. Our *in vitro* study revealed the negative effect of Aβ on insulin secretion, suggesting APP-derived Aβ may mediate the role of *APP* in pancreatic islets. Supporting this hypothesis, mice overexpressing APP exhibited Aβ accumulation in the pancreatic islets and decreased insulin levels in Aβ-positive regions (7). Additionally, Aβ deposition has been detected in the pancreas of subjects with T2D, leading us to speculate that peripheral Aβ might be a biomarker for β-cell dysfunction in human (8, 9). However, we did not find significant correlations between plasma Aβ40 and Aβ42 and HOMA-β among controls. This may be explained by the normal β-cell function in healthy controls, and we found significant and negative association of plasma Aβ40 with HOMA-β when combining both cases and controls (data not shown). Further epidemiological researches are needed to evaluate the forecast value of plasma Aβ for β-cell dysfunction.

Prediabetes is a metabolic intermediate state between normoglycemia and T2D, with 5-10% of prediabetic people progressing to T2D each year (15). Prediabetes is considered as a critical stage in preventing or delaying the onset of T2D due to its reversibility (15), therefore, it is important to understand the underlying mechanisms of prediabetes and to develop corresponding human biomarkers. This study, for the first time, reveals that plasma Aβ40 and Aβ42 are positively associated with prediabetes in human subjects. Similarly, our previous report demonstrated that higher plasma Aβ40 and Aβ42 levels were associated with increased risk of T2D (35). Previous case-control study also observed higher serum Aβ-autoantibody concentration, a biomarker reflecting Aβ level, in subjects with T2D (36). Yet, another case-control study reported that T2D cases exhibited lower plasma Aβ40 and Aβ42 levels than the controls (37). A plausible explanation for the inconsistency might be that the aforementioned study included cases with using hypoglycemic agents (37), which have been reported to affect plasma Aβ40 and Aβ42 levels (38–40). Therefore, the use of hypoglycemic agents should be considered when plasma Aβ is used as a biomarker for prediabetes and T2D.

Our study is the first that has combined *in vitro* studies and population-based studies to systematically explore the role of peripheral Aβ in insulin resistance, insulin secretion, and prediabetes. However, our study also has several limitations. Firstly, we were unable to determine the causality between plasma Aβ and prediabetes due to the observational nature of population-based studies. Secondly, the findings from population-based studies might be affected by residual confounding of other unmeasured factors, although we controlled for multiple risk factors of prediabetes. Thirdly, new-onset prediabetes was diagnosed only based on FPG in the Tongji-Ezhou cohort, and subjects who developed T2D might be misdiagnosed as prediabetes. Taken together with our previous study (35), plasm Aβ levels were not significantly different between prediabetes and T2D individuals. Hence, the positive association between plasma Aβ and new-onset prediabetes could persist if misdiagnosed subjects were excluded. Fourthly, glycated hemoglobin, an important parameter for glucose homeostasis, was not determined in our population-based studies. Fifthly, cognitive function was not evaluated in our population-based studies. Considering the positive associations of prediabetes and T2D with cognitive impairment and Alzheimer’s disease (41, 42), further studies are needed to explore the role of plasma Aβ in linking these conditions.

In conclusion, elevated plasma Aβ40 and Aβ42 levels were associated with increased risk of prediabetes in human subjects, which may be through impairing insulin sensitivity in hepatocytes and myotubes and insulin secretion in pancreatic β-cells. Plasma Aβ could be used as a predictor for prediabetes, and reducing plasma Aβ level may be a novel therapy for prediabetes. The molecular mechanisms of Aβ affecting peripheral insulin sensitivity and insulin secretion need to be further clarified.

## Data availability statement

The raw data supporting the conclusions of this article will be made available by the authors, without undue reservation.

## Ethics statement

The studies involving human participants were reviewed and approved by Ethics Committee of Tongji Medical College. The patients/participants provided their written informed consent to participate in this study.

## Author contributions

ZX, LGL, and XP designed the study. ZX, JC, PW, LYL, SH, HL, YH, XM, HY, ZS, DW, JX, and XP contributed to the acquisition, analysis, or interpretation of data. XP wrote the drafter of the paper. ZX, JC, PW, LYL, SH, HL, YH, XM, HY, ZS, DW, JX, and LGL contributed to reviewing and revising the paper. All authors read and approved the final manuscript.

## References

[1] Hampel H Hardy J Blennow K Chen C Perry G Kim SH. The amyloid-β Pathway in alzheimer's disease. Mol Psychiatry (2021) 26(10):5481–503. doi: 10.1038/s41380-021-01249-0 PMC8758495 34456336

[2] Lee YH Tharp WG Maple RL Nair S Permana PA Pratley RE. Amyloid precursor protein expression is upregulated in adipocytes in obesity. Obes (Silver Spring) (2008) 16(7):1493–500. doi: 10.1038/oby.2008.267 18483477

[3] Evin G Zhu A Holsinger RM Masters CL Li QX. Proteolytic processing of the alzheimer's disease amyloid precursor protein in brain and platelets. J Neurosci Res (2003) 74(3):386–92. doi: 10.1002/jnr.10745 14598315

[4] Kuo YM Kokjohn TA Watson MD Woods AS Cotter RJ Sue LI. Elevated abeta42 in skeletal muscle of alzheimer disease patients suggests peripheral alterations of abetapp metabolism. Am J Pathol (2000) 156(3):797–805. doi: 10.1016/s0002-9440(10)64947-4 10702395 PMC1876838

[5] Citron M Vigo-Pelfrey C Teplow DB Miller C Schenk D Johnston J. Excessive production of amyloid beta-protein by peripheral cells of symptomatic and presymptomatic patients carrying the swedish familial alzheimer disease mutation. Proc Natl Acad Sci U.S.A. (1994) 91(25):11993–7. doi: 10.1073/pnas.91.25.11993 PMC45362 7991571

[6] Hendrickx JO De Moudt S Calus E Martinet W Guns PDF Roth L. Serum corticosterone and insulin resistance as early biomarkers in the happ23 overexpressing mouse model of alzheimer's disease. Int J Mol Sci (2021) 22(13):6656. doi: 10.3390/ijms22136656 34206322 PMC8269119

[7] Vandal M White PJ Chevrier G Tremblay C St-Amour I Planel E. Age-dependent impairment of glucose tolerance in the 3xtg-ad mouse model of alzheimer's disease. FASEB J (2015) 29(10):4273–84. doi: 10.1096/fj.14-268482 26108977

[8] Martinez-Valbuena I Valenti-Azcarate R Amat-Villegas I Riverol M Marcilla I de Andrea CE. Amylin as a potential link between type 2 diabetes and alzheimer disease. Ann Neurol (2019) 86(4):539–51. doi: 10.1002/ana.25570 31376172

[9] Miklossy J Qing H Radenovic A Kis A Vileno B Laszlo F. Beta amyloid and hyperphosphorylated tau deposits in the pancreas in type 2 diabetes. Neurobiol Aging (2010) 31(9):1503–15. doi: 10.1016/j.neurobiolaging.2008.08.019 PMC4140193 18950899

[10] Vattemi G Engel WK McFerrin J Pastorino L Buxbaum JD Askanas V. Bace1 and bace2 in pathologic and normal human muscle. Exp Neurol (2003) 179(2):150–8. doi: 10.1016/s0014-4886(02)00025-0 12618121

[11] Roher AE Esh CL Kokjohn TA Castano EM Van Vickle GD Kalback WM. Amyloid beta peptides in human plasma and tissues and their significance for alzheimer's disease. Alzheimers Dement (2009) 5(1):18–29. doi: 10.1016/j.jalz.2008.10.004 19118806 PMC2663406

[12] Ferrannini E Gastaldelli A Iozzo P. Pathophysiology of prediabetes. Med Clin North Am (2011) 95(2):327–39 vii–viii. doi: 10.1016/j.mcna.2010.11.005 21281836

[13] Cai X Zhang Y Li M Wu JH Mai L Li J. Association between prediabetes and risk of all cause mortality and cardiovascular disease: updated meta-analysis. Bmj (2020) 370:m2297. doi: 10.1136/bmj.m2297 32669282 PMC7362233

[14] Huang Y Cai X Mai W Li M Hu Y. Association between prediabetes and risk of cardiovascular disease and all cause mortality: systematic review and meta-analysis. Bmj (2016) 355:i5953. doi: 10.1136/bmj.i5953 27881363 PMC5121106

[15] Tabák AG Herder C Rathmann W Brunner EJ Kivimäki M. Prediabetes: A high-risk state for diabetes development. Lancet (2012) 379(9833):2279–90. doi: 10.1016/s0140-6736(12)60283-9 PMC3891203 22683128

[16] Macklin L Griffith CM Cai Y Rose GM Yan XX Patrylo PR. Glucose tolerance and insulin sensitivity are impaired in app/ps1 transgenic mice prior to amyloid plaque pathogenesis and cognitive decline. Exp Gerontol (2017) 88:9–18. doi: 10.1016/j.exger.2016.12.019 28025127

[17] Zhang Y Zhou B Zhang F Wu J Hu Y Liu Y. Amyloid-beta induces hepatic insulin resistance by activating jak2/stat3/socs-1 signaling pathway. Diabetes (2012) 61(6):1434–43. doi: 10.2337/db11-0499 PMC3357286 22522613

[18] Chonpathompikunlert P Yoshitomi T Han J Isoda H Nagasaki Y. The use of nitroxide radical-containing nanoparticles coupled with piperine to protect neuroblastoma sh-sy5y cells from Aβ-induced oxidative stress. Biomaterials (2011) 32(33):8605–12. doi: 10.1016/j.biomaterials.2011.07.024 21855995

[19] Zhang Y Ranta F Tang C Shumilina E Mahmud H Föller M. Sphingomyelinase dependent apoptosis following treatment of pancreatic beta-cells with amyloid peptides abeta(1-42) or iapp. Apoptosis (2009) 14(7):878–89. doi: 10.1007/s10495-009-0364-4 19488858

[20] Baron P Galimberti D Meda L Prat E Scarpini E Conti G. Synergistic effect of beta-amyloid protein and interferon gamma on nitric oxide production by C2c12 muscle cells. Brain (2000) 123(Pt 2):374–9. doi: 10.1093/brain/123.2.374 10648444

[21] Yang YM Seo SY Kim TH Kim SG. Decrease of microrna-122 causes hepatic insulin resistance by inducing protein tyrosine phosphatase 1b, which is reversed by licorice flavonoid. Hepatology (2012) 56(6):2209–20. doi: 10.1002/hep.25912 22807119

[22] Wang X Feng Z Wang X Yang L Han S Cao K. O-glcnacase deficiency suppresses skeletal myogenesis and insulin sensitivity in mice through the modulation of mitochondrial homeostasis. Diabetologia (2016) 59(6):1287–96. doi: 10.1007/s00125-016-3919-2 26993634

[23] Haywood NJ Cordell PA Tang KY Makova N Yuldasheva NY Imrie H. Insulin-like growth factor binding protein 1 could improve glucose regulation and insulin sensitivity through its rgd domain. Diabetes (2017) 66(2):287–99. doi: 10.2337/db16-0997 28108607

[24] Alberti KG Zimmet PZ. Definition, diagnosis and classification of diabetes mellitus and its complications. Part 1: diagnosis and classification of diabetes mellitus provisional report of a who consultation. Diabetes Med (1998) 15(7):539–53. doi: 10.1002/(sici)1096-9136(199807)15:7<539::aid-dia668>3.0.co;2-s 9686693

[25] Song F Jia W Yao Y Hu Y Lei L Lin J. Oxidative stress, antioxidant status and DNA damage in patients with impaired glucose regulation and newly diagnosed type 2 diabetes. Clin Sci (2007) 112(12):599–606. doi: 10.1042/cs20060323 17209802

[26] Wijesekara N Ahrens R Sabale M Wu L Ha K Verdile G. Amyloid-beta and islet amyloid pathologies link alzheimer disease and type 2 diabetes in a transgenic model. FASEB J (2017) 31(12):5409–18. doi: 10.1096/fj.201700431R 28808140

[27] Zhang Y Zhou B Deng B Zhang F Wu J Wang Y. Amyloid-beta induces hepatic insulin resistance in vivo v*ia* jak2. Diabetes (2013) 62(4):1159–66. doi: 10.2337/db12-0670 PMC3609589 23223021

[28] Meakin PJ Harper AJ Hamilton DL Gallagher J McNeilly AD Burgess LA. Reduction in bace1 decreases body weight, protects against diet-induced obesity and enhances insulin sensitivity in mice. Biochem J (2012) 441(1):285–96. doi: 10.1042/bj20110512 PMC3242510 21880018

[29] Xie L Helmerhorst E Taddei K Plewright B Van Bronswijk W Martins R. Alzheimer's beta-amyloid peptides compete for insulin binding to the insulin receptor. J Neurosci (2002) 22(10):Rc221. doi: 10.1523/JNEUROSCI.22-10-j0001.2002 12006603 PMC6757630

[30] De Felice FG Vieira MN Bomfim TR Decker H Velasco PT Lambert MP. Protection of synapses against alzheimer's-linked toxins: insulin signaling prevents the pathogenic binding of abeta oligomers. Proc Natl Acad Sci U.S.A. (2009) 106(6):1971–6. doi: 10.1073/pnas.0809158106 PMC2634809 19188609

[31] Zhao WQ De Felice FG Fernandez S Chen H Lambert MP Quon MJ. Amyloid beta oligomers induce impairment of neuronal insulin receptors. FASEB J (2008) 22(1):246–60. doi: 10.1096/fj.06-7703com 17720802

[32] Lourenco MV Clarke JR Frozza RL Bomfim TR Forny-Germano L Batista AF. Tnf-alpha mediates pkr-dependent memory impairment and brain irs-1 inhibition induced by alzheimer's beta-amyloid oligomers in mice and monkeys. Cell Metab (2013) 18(6):831–43. doi: 10.1016/j.cmet.2013.11.002 24315369

[33] Bomfim TR Forny-Germano L Sathler LB Brito-Moreira J Houzel JC Decker H. An anti-diabetes agent protects the mouse brain from defective insulin signaling caused by alzheimer's disease- associated abeta oligomers. J Clin Invest (2012) 122(4):1339–53. doi: 10.1172/jci57256 PMC3314445 22476196

[34] Tu Z Keller MP Zhang C Rabaglia ME Greenawalt DM Yang X. Integrative analysis of a cross-loci regulation network identifies app as a gene regulating insulin secretion from pancreatic islets. PloS Genet (2012) 8(12):e1003107. doi: 10.1371/journal.pgen.1003107 23236292 PMC3516550

[35] Peng X Xu Z Mo X Guo Q Yin J Xu M. Association of plasma β-amyloid 40 and 42 concentration with type 2 diabetes among chinese adults. Diabetologia (2020) 63(5):954–63. doi: 10.1007/s00125-020-05102-x 32034441

[36] Kim I Lee J Hong HJ Jung ES Ku YH Jeong IK. A Relationship between Alzheimer's Disease and Type 2 Diabetes Mellitus through the Measurement of Serum Amyloid-Beta Autoantibodies. J Alzheimers Dis (2010) 19(4):1371–6. doi: 10.3233/jad-2010-1332 20061608

[37] Peters KE Davis WA Taddei K Martins RN Masters CL Davis TM. Plasma amyloid-beta peptides in type 2 diabetes: A matched case-control ctudy. J Alzheimers Dis (2017) 56(3):1127–33. doi: 10.3233/jad-161050 28106562

[38] Stanley M Macauley SL Caesar EE Koscal LJ Moritz W Robinson GO. The effects of peripheral and central high insulin on brain insulin signaling and amyloid-beta in young and old app/ps1 mice. J Neurosci (2016) 36(46):11704–15. doi: 10.1523/jneurosci.2119-16.2016 PMC5125227 27852778

[39] Swaminathan SK Ahlschwede KM Sarma V Curran GL Omtri RS Decklever T. Insulin differentially affects the distribution kinetics of amyloid beta 40 and 42 in plasma and brain. J Cereb Blood Flow Metab (2017) 38(5):904–18. doi: 10.1177/0271678X17709709 PMC5987944 28569090

[40] Tamaki C Ohtsuki S Terasaki T. Insulin facilitates the hepatic clearance of plasma amyloid beta-peptide (1-40) by intracellular translocation of low-density lipoprotein receptor-related protein 1 (Lrp-1) to the plasma membrane in hepatocytes. Mol Pharmacol (2007) 72(4):850–5. doi: 10.1124/mol.107.036913 17609417

[41] Xue M Xu W Ou YN Cao XP Tan MS Tan L. Diabetes mellitus and risks of cognitive impairment and dementia: A systematic review and meta-analysis of 144 prospective studies. Ageing Res Rev (2019) 55:100944. doi: 10.1016/j.arr.2019.100944 31430566

[42] Mallorquí-Bagué N Lozano-Madrid M Toledo E Corella D Salas-Salvadó J Cuenca-Royo A. Type 2 diabetes and cognitive impairment in an older population with overweight or obesity and metabolic syndrome: baseline cross-sectional analysis of the predimed-plus study. Sci Rep (2018) 8(1):16128. doi: 10.1038/s41598-018-33843-8 30382190 PMC6208341

