# Supplementary material for: The role of peripheral β-amyloid in insulin resistance, insulin secretion, and prediabetes: in vitro and population-based studies
Source: Front Endocrinol (Lausanne). 2023 Jul 19;14:1195658. doi: 10.3389/fendo.2023.1195658 (PMC10394827; doi:10.3389/fendo.2023.1195658)
Supplement: Supplementary file 1 [file DataSheet_1.docx]

Supplementary Material

The role of peripheral β-amyloid in insulin resistance, insulin secretion, and prediabetes: in vitro and population-based studies

**Zihui Xu****^1,2^, Juan Chen^1,2^, Pei Wang^1,2^, Linyan Li^1,2^, Shan Hu^1,2^, Hongjie Liu^1,2^, Yue Huang^1,2^, Xiaoxing Mo^1,2^, Hong Yan^2^, Zhilei Shan^1,2^, Di Wang^3^, Jian Xu^4^, Liegang Liu^1,2^, Xiaobo Peng^1,2*^**

*** Correspondence:** Xiaobo Peng: [xiaobopeng92@hust.edu.cn](mailto:xiaobopeng92@hust.edu.cn)

# Supplementary Figures and Tables

**Supplemental Table 1**Spearman correlations of plasma Aβ40 and Aβ42 with parameters of glucose metabolism among healthy participants in the case-control study

**Supplemental Fig. 1** Effects of Aβ on the viability of HepG2 cells. HepG2 cells were treated with the indicated concentrations of Aβ40 (a) or Aβ42 (b) for 12, 24, and 48 h, and then cell viability was detected by CCK-8 assays (n = 3). Data were presented as mean ± SEM. ^*^*p* <0.05, ^**^*p* <0.01, and ^***^*p* <0.001 for Aβ treated cells versus control cells at the same intervention time.

**Supplemental Fig. 2** Effects of Aβ on the viability of C2C12 myotubes. C2C12 myotubes were treated with the indicated concentrations of Aβ40 (a) or Aβ42 (b) for 12, 24, and 48 h, and then cell viability was detected by CCK-8 assays (n = 3). Data were presented as mean ± SEM. ^*^*p* <0.05, ^**^*p* <0.01, and ^***^*p* <0.001 for Aβ treated cells versus control cells at the same intervention time.

**Supplemental Fig. 3** Effects of Aβ on the viability of INS-1 cells. INS-1 cells were treated with the indicated concentrations of Aβ40 (a) or Aβ42 (b) for 12, 24, and 48 h, and then cell viability was detected by CCK-8 assays (n = 3). Data were presented as mean ± SEM. ^*^*p* <0.05, ^**^*p* <0.01, and ^***^*p* <0.001 for Aβ treated cells versus control cells at the same intervention time.

**Supplemental Table 1** Spearman correlations of plasma Aβ40 and Aβ42 with parameters of glucose metabolism among healthy participants in the case-control study

| Variables | FPG | FPI | HOMA-IR | HOMA-β | TyG index |
| --- | --- | --- | --- | --- | --- |
| Aβ40 |  |  |  |  |  |
| Model^a^ |  |  |  |  |  |
| Crude | 0.189^d^ | 0.090 | 0.137^b^ | -0.073 | 0.125^c^ |
| Model 1 | 0.226^d^ | 0.127^b^ | 0.176^c^ | -0.052 | 0.198^d^ |
| Model 2 | 0.220^d^ | 0.128^b^ | 0.175^c^ | -0.045 | 0.204^d^ |
| Aβ42 |  |  |  |  |  |
| Model^a^ |  |  |  |  |  |
| Crude | 0.090^b^ | 0.056 | 0.087 | -0.045 | 0.026 |
| Model 1 | 0.146^d^ | 0.080 | 0.116^b^ | -0.041 | 0.105^b^ |
| Model 2 | 0.145^d^ | 0.078 | 0.114^b^ | -0.043 | 0.106^b^ |

^a^ Model 1 was adjusted for age, sex, and BMI. Model 2 was additionally adjusted for current smoking status, current drinking status, physical activity, history of hypertension, history of CVD, and family history of diabetes.

^b^ *p* <0.05

^c^ *p* <0.01

^d^ *p* <0.001

**
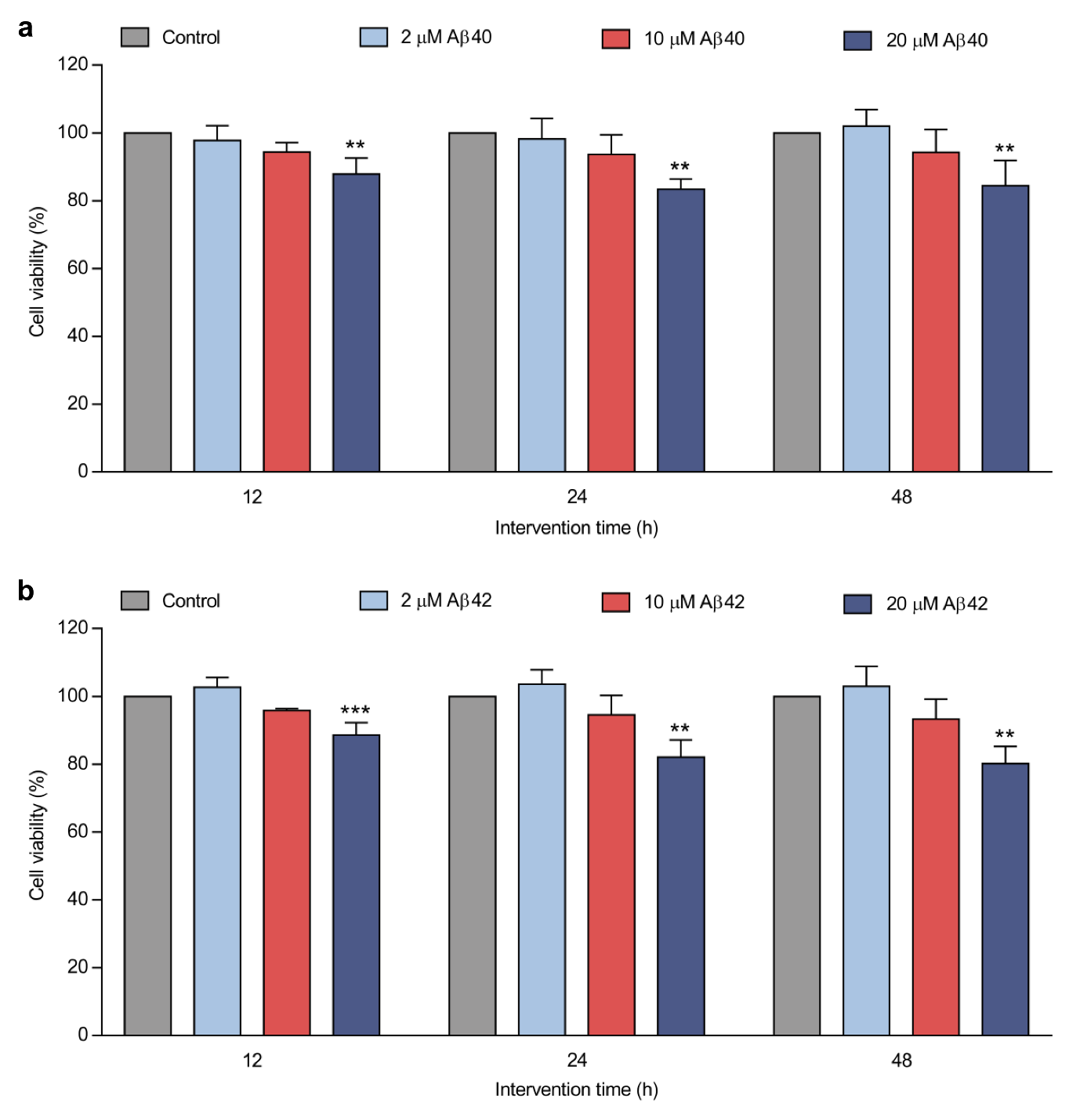
**

**Supplemental Fig. 1**Effects of Aβ on the viability of HepG2 cells. HepG2 cells were treated with the indicated concentrations of Aβ40 (a) or Aβ42 (b) for 12, 24, and 48 h, and then cell viability was detected by CCK-8 assays (*n* = 3). Data were presented as mean ± SEM. ^*^*p* <0.05, ^**^*p* <0.01, and ^***^*p* <0.001 for Aβ treated cells versus control cells at the same intervention time.


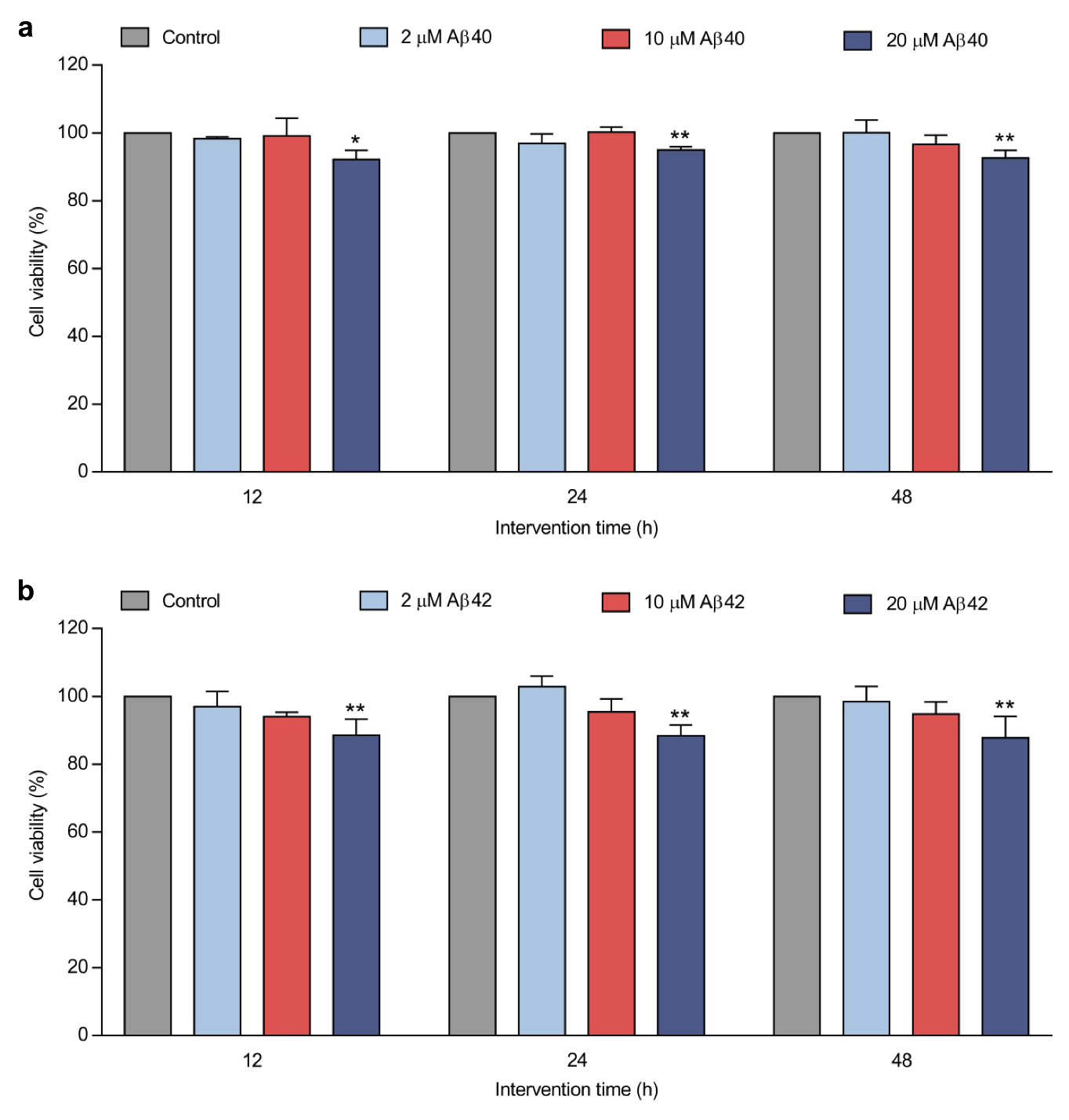


**Supplemental Fig. 2** Effects of Aβ on the viability of C2C12 myotubes. C2C12 myotubes were treated with the indicated concentrations of Aβ40 (a) or Aβ42 (b) for 12, 24, and 48 h, and then cell viability was detected by CCK-8 assays (n = 3). Data were presented as mean ± SEM. ^*^*p* <0.05, ^**^*p* <0.01, and ^***^*p* <0.001 for Aβ treated cells versus control cells at the same intervention time.


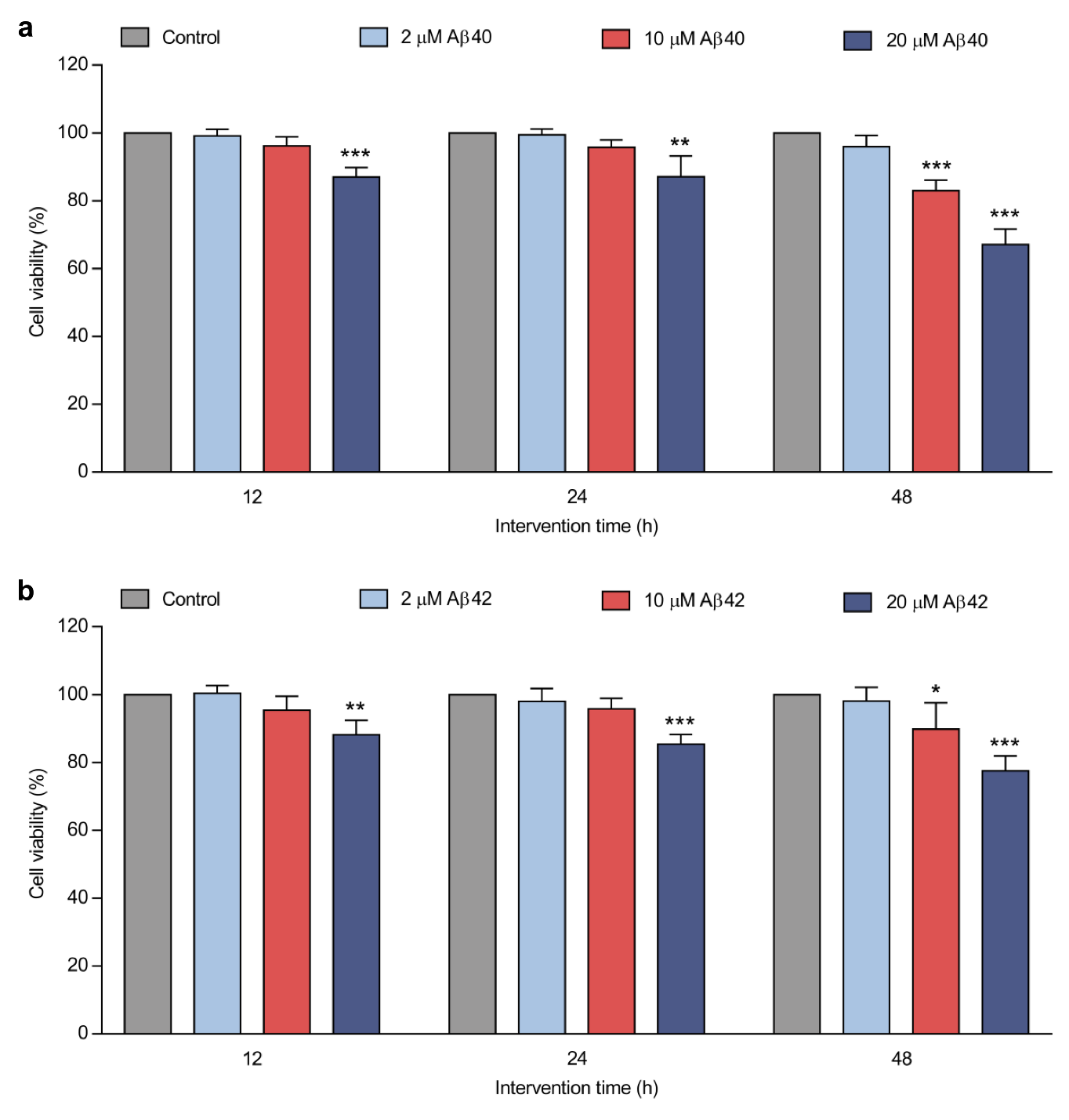


**Supplemental Fig. 3** Effects of Aβ on the viability of INS-1 cells. INS-1 cells were treated with the indicated concentrations of Aβ40 (a) or Aβ42 (b) for 12, 24, and 48 h, and then cell viability was detected by CCK-8 assays (n = 3). Data were presented as mean ± SEM. ^*^*p* <0.05, ^**^*p* <0.01, and ^***^*p* <0.001 for Aβ treated cells versus control cells at the same intervention time.
